# Breaking the Mold: Transcription Factors in the Anucleate Platelet and Platelet-Derived Microparticles

**DOI:** 10.3389/fimmu.2015.00048

**Published:** 2015-02-13

**Authors:** Katie L. Lannan, Julie Sahler, Nina Kim, Sherry L. Spinelli, Sanjay B. Maggirwar, Olivier Garraud, Fabrice Cognasse, Neil Blumberg, Richard P. Phipps

**Affiliations:** ^1^Department of Microbiology and Immunology, University of Rochester School of Medicine and Dentistry, Rochester, NY, USA; ^2^Department of Biological and Environmental Engineering, Cornell University, Ithaca, NY, USA; ^3^Department of Pathology and Laboratory Medicine, University of Rochester School of Medicine and Dentistry, Rochester, NY, USA; ^4^Faculté de Médecine, Université de Lyon, Saint-Etienne, France; ^5^Etablissement Français du Sang Auvergne-Loire, Saint-Etienne, France; ^6^Department of Environmental Medicine, University of Rochester School of Medicine and Dentistry, Rochester, NY, USA

**Keywords:** platelets, microparticles, transcription factors, NF-kappa B, PPAR gamma, non-genomic, PPAR alpha, steroid receptors

## Abstract

Platelets are small anucleate blood cells derived from megakaryocytes. In addition to their pivotal roles in hemostasis, platelets are the smallest, yet most abundant, immune cells and regulate inflammation, immunity, and disease progression. Although platelets lack DNA, and thus no functional transcriptional activities, they are nonetheless rich sources of RNAs, possess an intact spliceosome, and are thus capable of synthesizing proteins. Previously, it was thought that platelet RNAs and translational machinery were remnants from the megakaryocyte. We now know that the initial description of platelets as “cellular fragments” is an antiquated notion, as mounting evidence suggests otherwise. Therefore, it is reasonable to hypothesize that platelet transcription factors are not vestigial remnants from megakaryocytes, but have important, if only partly understood functions. Proteins play multiple cellular roles to minimize energy expenditure for maximum cellular function; thus, the same can be expected for transcription factors. In fact, numerous transcription factors have non-genomic roles, both in platelets and in nucleated cells. Our lab and others have discovered the presence and non-genomic roles of transcription factors in platelets, such as the nuclear factor kappa β (NFκB) family of proteins and peroxisome proliferator-activated receptor gamma (PPARγ). In addition to numerous roles in regulating platelet activation, functional transcription factors can be transferred to vascular and immune cells through platelet microparticles. This method of transcellular delivery of key immune molecules may be a vital mechanism by which platelet transcription factors regulate inflammation and immunity. At the very least, platelets are an ideal model cell to dissect out the non-genomic roles of transcription factors in nucleated cells. There is abundant evidence to suggest that transcription factors in platelets play key roles in regulating inflammatory and hemostatic functions.

## Introduction

Platelets are central players in hemostasis and inflammation, contributing to numerous pathophysiologic conditions (1). They are unique from the majority of mammalian cells apart from red blood cells in that they lack a nucleus, and thus have previously been discounted as “cellular fragments.” This antiquated notion has been refuted many times over as platelets are now emerging as cellular mediators of cancer cell metastasis, atherosclerosis, type II diabetes, and even mediate adaptive immune responses (2, 3). Platelets are metabolically active cells that contain numerous functional organelles, such as endoplasmic reticulum, Golgi apparatus, mitochondria, and granules that can be released upon activation. Although their lack of a nucleus prevents *de novo* transcription, they can be activated very rapidly to release copious amounts of biological mediators within seconds to minutes of stimulation. The idea that platelets contain transcription factors is a relatively new concept and has led to the discovery of a large number of transcription factors in platelets (Table 1). This review will discuss the newly described roles of transcription factors in platelets, in addition to proposing uninvestigated potential roles of transcription factors in platelets, as extrapolated from findings in nucleated cells (Table 2).

**Table 1:** **Identified transcription factors in platelets**.

Transcription factor	Activation of transcription factor	Agonist-induced activation
p65	Phosphorylation	↑Aggregation, spreading, clot retraction, GPIBα shedding
PPARγ	Ligand binding (15d-PGJ_2_, TZDs)	↓Aggregation, CD40L, TXB_2_, P-selectin
	Phosphorylation	↑Collagen-induced activation and granule secretion
PPARβ/δ	Ligand binding (GW501516, PGI_2_)	↓Aggregation
PPARα	Ligand binding (fenofibrate)	↓Aggregation
	Phosphorylation	↑Activation
LXRβ	Ligand binding	↓Collagen-induced aggregation
RXRα	Ligand binding (9cRA)	↓Activation
GR	Ligand binding (prednisolone)	↓Activation
	Ligand binding (dexamethasone)	Unknown
AHR	Ligand binding	↑Activation
STAT3	Phosphorylation	↑Aggregation, P-selectin, thrombosis

**Table 2:** **Identified and possible interactions of transcription factors in platelets with other proteins**.

Transcription factor	Known protein interaction		Possible protein interactions	
	Binding partners	Outcome	Binding partners	Outcome
PPARγ	Syk, LAT	Platelet activation	NFκB (p65)	Inhibit NFκB activity → anti-platelet effect
	p-ERK, p-p38	Granule secretion	MEK/ERK	Increased activation
	PKCα	Reduce PKCα activation	
PPARβ/δ	PKCα	Dampen platelet adhesion	–	–
PPARα	NFkB	Inhibit NFκB activity → anti-platelet effect	PKCα	Increased activation
LXRβ	Syk, LAT	Dampen platelet activation	PPARγ	Unknown
RXRα	PPARγ	Unknown	G_q_	Decreased activation
GR	HSP90	Unknown	–	–
	Mineralocorticoid Receptor	Unknown	–	–
AHR	–	–	NFκB (p65)	Unknown

## Nuclear Factor Kappa β

In the Immunology field, nuclear factor kappa β (NFκB) is the most widely recognized transcription factor for its quintessential roles in regulating inflammation and immune responses. Almost any immunologist could rattle off key parts of its signaling pathways in response to toll-like receptor (TLR) signaling, influenza infection, or in cytokine production. Although we are quick to identify its essential roles in regulating transcription of inflammatory genes, the non-genomic roles of NFκB are often overlooked. In all fairness, the concept that NFκB has non-genomic roles in nucleated and non-nucleated cells is a relatively new area of study that is still in its early stages (4). NFκB signaling molecules regulate several different stages of the inflammatory response, without ever entering the nucleus. For example, the NFκB regulatory protein, IkappaB kinase β (IKKβ), can alter the function of numerous proteins via phosphorylation in addition to regulating signaling through direct interactions with cellular effector molecules (5).

## IkappaB Kinase in Platelets

Specifically in platelets, the presence and non-genomic functions of NFκB family members have been demonstrated by several groups, including our own (6–9). Our group discovered the presence of the majority of NFκB family members in human platelets, including the canonical p50/p65 subunits, RelB and c-Rel. Additionally, we identified the presence of IκB proteins and IKK members, which regulate NFκB activation (6). Importantly, these findings suggest that platelets contain an intact, functional, and complete NFκB pathway. The use of the irreversible inhibitor of IKKβ phosphorylation, BAY 11-7082 (BAY), has elucidated complex roles for NFκB in platelet signaling. In human platelets, BAY inhibits platelet spreading and clot retraction, and may alter aggregation at higher doses (6, 10, 11). Malaver et al. (7) and Chen et al. (12) show that high concentrations of BAY (10–25 μM) inhibit platelet aggregation, while our group saw no effect when platelets were treated with 0.5–5 μM BAY (6). As the IC_50_ of BAY for inhibition of IKK-mediated phosphorylation of IκBα is 10 μM, <5 μM BAY may be too low to inhibit IKK sufficiently to affect aggregation (13). Another possibility is that IKK inhibition exerts a threshold response in affecting platelet aggregation, rather than a dose–response. Using genetic approaches, Karim et al. demonstrated that IKKβ knockout mice have variable, but generally attenuated, aggregation responses to platelet agonists, which may explain some of the variations observed in human studies as well (14).

Interestingly, the data reported by Chen et al. and Malavar et al. suggest that the second wave of aggregation was most affected by IKKβ inhibition, thus affecting the maximum aggregation and potentiation of the response in human platelets (7, 12). In accordance with this finding, dense granule release was consistently and potently reduced by IKKβ inhibition in these and other studies. This finding is likely explained by the ability of IKKβ to phosphorylate synaptosomal-associated protein-23 (SNAP-23), an integral regulator of granule secretion (14). SNAP-23 is a member of the target sensitive factor attachment protein receptors (SNARE) complex, which interacts with the vesicle-associated membrane protein (VAMP) to facilitate granule release. IKKβ appears to be central in enhancing soluble *N*-ethylmaleimide–SNARE complex formation though the phosphorylation of SNAP-23. Consequently, platelets from IKKβ knockout mice have a reduced ability to release alpha, dense, and lysosomal granules upon thrombin stimulation by approximately 30%. Likewise, treatment of mouse or human platelets with an inhibitor of IKKβ similarly reduced granule secretion upon stimulation. These data suggest that the NFκB signaling molecule, IKKβ, plays an important regulatory role in platelet activation by transducing critical stimulatory signals.

In general, genetic ablation or pharmacological inhibition of IKKβ in mice results in a hyporesponsive phenotype to agonist-induced platelet activation. Inhibition of IKKβ in mice prolonged thrombus formation and increased bleeding times (14). Additionally, a second IKKβ inhibitor, IKK inhibitor VII, recapitulated several of the aforementioned findings in both human and mouse platelets, including preventing P-selectin expression and dampening aggregation (15). Of note, Gambaryan et al. found that IKK inhibitor VII potentiated collagen and thrombin-induced aggregation, rather than having an inhibitory effect (8). They proposed a model in which thrombin and collagen induce a negative feedback loop in platelets that inhibits platelet function through NFκB. Although the work delves into the complex agonist-induced signaling pathways of NFκB in platelets, their data showing potentiation of platelet activation by IKK inhibitor VII is less clear-cut. Treatment of human platelets with IKK inhibitor VII enhanced very low dose (0.001 U/mL) thrombin-induced PAC1 binding by approximately 15%. Additionally, IKK inhibitor VII treatment only mildly potentiated collagen (10 μg/mL) and thrombin (0.01 U/mL)-induced platelet aggregation, with a slight left shift in the aggregation traces compared to control. However, the maximum amplitude of aggregation was indistinguishable between IKK inhibitor VII treatment and control. Although the data demonstrating NFκB signaling post-activation are intriguing, the current consensus is that NFκB primarily plays an important role in positively regulating platelet activation.

As much of the investigations into the non-genomic roles of the transcription factor, NFκB, has involved manipulation of its upstream regulatory kinase, IKK, careful consideration must be taken into account when interpreting the findings of these studies. IKK acts as a kinase that plays a crucial role in regulating NFκB activation, but can also phosphorylate other proteins that may play regulatory roles in platelet activation (5). Thus, inhibition of IKK in platelets may dampen platelet function in a non-canonical fashion, independent of NFκB. On the other hand, many studies have observed changes in p65 phosphorylation in platelets, although the effects of direct inhibition or deletion of p65 has not been investigated to date. It is likely that IKK activation in platelets results in NFκB-dependent and -independent regulation of platelet function. This is supported by the data showing that IKK phosphorylates SNAP-23 (14), leading to granule secretion, but can also activate p65, which can regulate protein kinase A (PKA) (8).

## NFκB in Platelets during Inflammation

Nuclear factor kappa β in nucleated cells is known to play a crucial role in inflammatory diseases, although its functions in platelets during inflammation are still under active investigation. In one study, IKKβ deficiency increased neointimal formation in low-density lipoprotein receptor (LDLR) knockout mice and exhibited increased leukocyte adherence to the vessel walls after injury (16). Upon further investigation, IKKβ-deficient platelets were unable to shed GPIbα in response to thrombin stimulation. Interestingly, GPIbα shedding in response to ADP or collagen was not affected, suggesting that IKKβ is uniquely involved in thrombin-induced GPIbα shedding. These data are intriguing in light of the fact that many studies evaluating the role of IKKβ in platelets focused on thrombin-induced activation and signaling. However, collagen and ADP-induced aggregation and granule secretion were also dampened by IKKβ deficiency or pharmacological inhibition. Furthermore, no differences in GPVI, GPIX, or αIIbβ3 shedding were found by loss of IKKβ in mouse platelets. These data suggest that although IKKβ plays an important role in the activation of platelets, it may also induce an inhibitory feedback loop, as proposed by Gambaryan et al., perhaps through shedding of GPIbα (8). Sustained levels of GPIbα on the platelet surface can enhance platelet–leukocyte interactions, and thus, may exacerbate certain conditions.

Platelets are also known to response to various immunologic stimuli, such as TLR ligands (17). In nucleated cells, bacterial lipopolysaccharide (LPS) signaling through TLR4 is largely through NFκB, leading to the production of proinflammatory cytokines and chemokines. We have recently shown that platelets can discriminate between different isoforms of LPS (18). This suggests that platelets are capable of specifically sensing and responding to various bacterial products. Thus, it is possible that LPS signaling in platelets involves differential NFkB activation, although this has not been investigated in platelets to date.

## Signaling Mechanism of NFκB in Platelets

Thrombin activates the NFκB signaling cascade in platelets, although the complete pathway has not yet been elucidated. Several lines of evidence suggest that p38 mitogen-activated protein kinase (MAPK) signaling is upstream of NFκB in platelets, while the extracellular signal-regulated kinase (ERK) pathway is downstream of NFκB activation (19, 20). In human platelets, inhibition of MAPK prevented collagen-induced IKKβ and p65 phosphorylation, while treatment with an ERK inhibitor had no effect. Furthermore, collagen-induced ERK phosphorylation was prevented by pretreatment with an MAPK inhibitor or the NFκB inhibitor BAY, suggesting that NFκB signaling regulates ERK activation. ERK activates phospholipase A_2_ (PLA_2_), which releases arachidonic acid, leading to platelet aggregation and mediator release. This explains why arachidonic acid-induced platelet aggregation is unaffected by NFκB inhibitors, while aggregation in response to other agonists is dampened. Interestingly, one study suggests that activation of NFκB by CD40L signaling may be independent of p38 MAPK, but may instead involve TRAF2 activation of IKKβ (15). Additionally, protease-activated receptor 4 (PAR4) stimulation led to ceramide production by sphingomyelin phosphodiesterase (Smase), which in turn activated MAPK, while PAR1 signaling was independent of ceramide (12). This is consistent with the finding that exogenous treatment of platelets with ceramide leads to *in vitro* activation and enhances thrombosis *in vivo*. PAR1 and PAR4 are the thrombin receptors on human platelets, with PAR1 having a lower threshold for activation by thrombin than PAR4 (21). PAR1 activation typically induces a rapid, but transient spike in calcium, while PAR4 activation involves a more sustained response, suggesting overlapping, but distinct roles for these receptors (22). These data reveal a novel and distinct signaling pathway for PAR1 and PAR4 receptors, although both converge on NFκB signaling.

**Figure 1:**
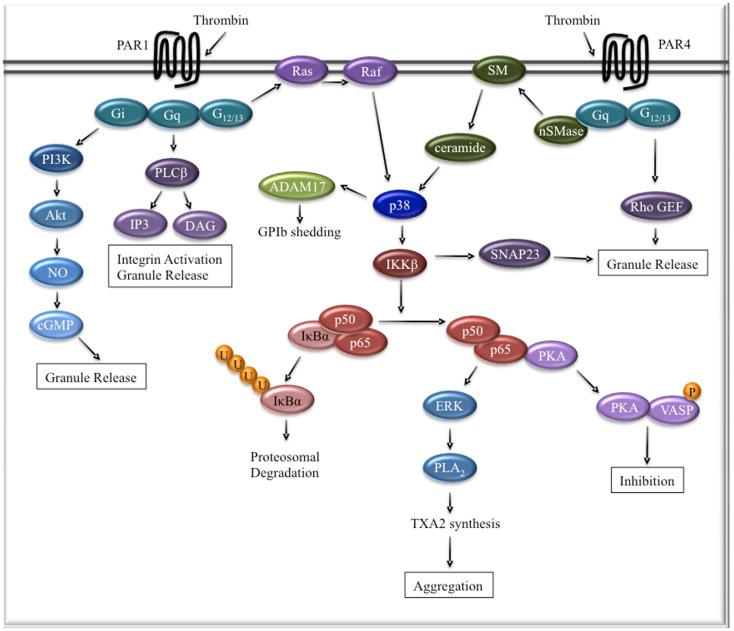
**The role of NFκB in platelet thrombin signaling**. Thrombin signaling through PAR1 in human platelets is mostly independent of NFκB. PAR4 signaling in human platelets involves activation of p38 MAPK through ceramide signaling. Activated p38 MAPK phosphorylates IKKβ, leading to the subsequent ubiquitination and proteosomal degradation of the inhibitory IκBα protein. Active p50/p65 dimers activate the ERK signaling cascade, which is involved in thromboxane TXA_2_ synthesis and aggregation. The p65 subunit can also bind PKA, inducing a negative feedback loop through VASP phosphorylation.

Taken together, these data present compelling evidence that NFκB plays an important, albeit complex, role in platelet activation (Figure 1). The data support a model whereby platelet activation through various receptors leads to phosphorylation and activation of IKKβ, release of p65, and subsequent platelet aggregation and granule release. This model, however, is not mutually exclusive of the idea that NFκB may induce a negative inhibitory feedback loop in platelets (8). After dissociation of p65 from its inhibitory complex, PKA is free to induce vasodilator-stimulated phosphoprotein (VASP) phosphorylation, which mediates platelet inhibitory signaling. This may represent a mechanism to fine tune platelet activation after thrombin stimulation. In fact, PIP_3_ can induce VASP phosphorylation, leading to inhibitory signaling, in addition to activating IKK through protein kinase B, also known as Akt. Thrombin signaling also appears to be unique in that it stimulates GPIb shedding through NFκB and ADAM17, unlike ADP or collagen-induced activation (16). ADAM17 is a sheddase that is critical for platelet surface receptor shedding and can be activated by p38 MAPK (23). Unlike pharmacological inhibition of NFκB in human platelets, IKK-deficient mouse platelets are unable to phosphorylate p38 MAPK after thrombin stimulation (16). This raises the question as to whether defective GPIb shedding in IKKβ-deficient mouse platelets is an artifact of interspecies variability, differences in inhibition versus complete lack of IKKβ, or merely a technical timing issue. Regardless, it will be necessary to investigate the role of ADAM17 and GPIb shedding in human platelet NFκB signaling.

## Non-Genomic Functions of NFκB: Lessons from Nucleated Cells

Nuclear factor kappa β is a versatile family of proteins capable of performing multiple functions in nucleated cells apart from acting as a transcription factor (5). Evaluating these identified non-genomic roles in nucleated cells is likely to translate to important regulatory functions in platelets. Although roles for IKKβ in platelets have already been identified, studies from nucleated cells suggest that there may be more. A strong contender is the role of IKKβ in platelet spreading, as inhibition of IKKβ in human platelets leads to a spreading defect. In epithelial cells and B-lymphocytes, IKKβ-mediated phosphorylation of Dok1 inhibits ERK activation, leading to increased cell motility (24). Platelets are known to transiently activate ERK, which is important for alpha granule release. However, outside-in integrin signaling through the fibrinogen receptor, as is likely to occur during platelet spreading, inhibits ERK signaling (25). Thus, inhibiting IKKβ may impair platelet spreading through a similar Dok1-mediated mechanism. Proteomic data have identified the presence of Dok1 in human platelets, although its function has yet to be studied in this context (26).

The NFκB protein p65 and IκBα, are found in the mitochondria and appear to be differentially regulated compared to their cytoplasmic counterparts (27). Interestingly, canonical NFκB stimulatory signals had no effect on the expression or phosphorylation of mitochondria-localized p65 (28). Conversely, stimulation of liver or Jurkat cells with tumor necrosis factor α (TNFα) or FAS ligand, respectively, led to non-proteasomal degradation of IκBα and induction of apoptosis. IKKβ, on the other hand, was not localized to the mitochondria. To date, most studies of NFκB in platelets have involved the use of IKKβ inhibitors and IKKβ knockout mice. Investigations in platelets centering on the role of IκBα and p65, in lieu of IKKβ, may reveal novel apoptotic regulatory elements in platelets.

A second intriguing possibility is the role of 14–3–3β in regulating mRNA stability in platelets. 14–3–3β is a highly conserved protein that binds AU-rich elements (ARE) on mRNA, leading to destabilization of mRNA (29). Phosphorylation of 14–3–3β by IKK results in the release of a 14–3–3β-tritetraprolin complex from the mRNA, thus preventing its destabilizing effects (30). This is interesting in light of the fact that platelets contain mRNA, which can be translated upon stimulation (31) Furthermore, 14–3–3 proteins can interact with p65 and IκBα to facilitate the export of p65 from the nucleus. As proteomic data show that human platelets express 14–3–3β (32), it is possible that it plays an important role in regulating mRNA stability or NFκB signaling in the anucleate platelet.

## PPARγ in Platelets

Peroxisome proliferator-activated receptor γ (PPARγ) is a ligand-activated transcription factor that is important in the regulation of lipid and glucose metabolism and is essential for fat production. Inactive PPARγ is localized to the cytoplasm of nucleated cells complexed with co-repressors. Upon ligand binding, the co-repressors dissociate, leading to a conformational change in PPARγ, allowing dimerization with retinoid X receptor α (RXRα) (33). This heterodimeric complex then acts as a scaffold for the recruitment of coactivator proteins, and PPARγ-dependent gene transcription commences. Our group discovered that human and mouse platelets express functional PPARγ protein that is regulated by activation or treatment with PPARγ ligands (34).

## PPARγ Ligands are Cardioprotective

Thiazolidines (TZDs) are a class of oral antidiabetic drugs that exert their insulin-sensitizing actions through activation of PPARγ. In addition to regulating glucose homeostasis, TZDs, such as rosiglitazone, pioglitazone, and troglitazone, have potent anti-inflammatory properties (35). Type II diabetics taking TZDs have improved glucose metabolism, decreased markers of inflammation, and improved cardiovascular health. Much of this can be attributed to the beneficial actions of TZDs on vascular cells and both direct and indirect actions on circulating platelets. Platelets from type II diabetics exhibit a more activated phenotype and are hyper-responsive to agonist (36, 37). These changes include increased platelet numbers and mean platelet volume (MPV), which may indicate alterations in megakaryocyte function or increased platelet turnover (38). Additionally, platelets from type II diabetics have enhanced surface expression of the collagen receptor (GPVI) and the fibrinogen receptor (αIIbβ3), leading to increased adhesiveness and aggregation (39). Plasma from type II diabetics contains higher levels of the platelet-derived inflammatory mediators, soluble CD40L (sCD40L), soluble P-selectin (sP-selectin), and C-reactive protein (40, 41). Dsyregulated platelet function in type II diabetics is likely due to both inherent changes in the platelet and decreased prostaglandin I_2_ (PGI_2_) and nitric oxide (NO) production from endothelial cells, which exert potent anti-platelet effects.

Numerous studies have demonstrated that TZDs beneficially regulate cardiovascular function in type II diabetics. They have consistently been shown to reduce the elevated levels of plasminogen activator inhibitor 1 (PAI-1) (42–44). PAI-1 rapidly binds and inactivates tissue plasminogen activator, thus preventing fibrinolysis and increasing thrombotic risk. Additionally, TZDs reduce markers of cardiovascular disease, which are typically elevated in type II diabetics, including C-reactive protein, serum amyloid A, fibrinogen, and matrix metalloproteinase 9 (45–49). Many of these beneficial changes can be attributed to improved glucose metabolism and restored vascular and endothelial homeostasis, although similar results can be seen in non-diabetic patients (50).

## PPARγ Ligands Improve Platelet Function in Type II Diabetic

Consistent with their ability to reduce thrombotic risk, TZDs reduce markers of platelet activation. Rosiglitazone monotherapy decreased plasma sCD40L and P-selectin levels (51, 52). These data suggest that TZDs can directly or indirectly reduce platelet activation in type II diabetics. Supporting these findings, Khanolkar et al. demonstrated that patients receiving rosiglitazone and metformin had a significantly greater reduction in platelet aggregation compared to patients receiving gliclazide and metformin (53). Similarly, a second study found that pioglitazone in combination with metformin improved platelet function to a greater degree than glimepriride and metformin (49). A major unanswered question from these studies is whether TZDs improve cardiovascular health secondary to improved lipid metabolism or due to the anti-inflammatory actions of PPARγ stimulation. For example, macrophages, which play a key role in atherosclerosis, efflux cholesterol and are less inflammatory in response to PPARγ activation. In an attempt to investigate the insulin-sensitizing independent effects of TZDs, non-diabetic patients with coronary artery disease were treated with TZDs for 12 weeks. In this setting, TZDs reduced the inflammatory markers, C-reactive protein, tumor necrosis factor α (TNFα), and interleukin-6 (IL-6) (50, 54). Additionally, circulating platelet activity was dampened, as evidenced by fewer P-selectin positive platelets, suggesting an important role for the anti-inflammatory actions of PPARγ.

## PPARγ Ligands Dampen Platelet Function from Healthy Donors

Due to the global effects of TZDs, it is difficult to tease out indirect actions from potential direct effects of TZDs on platelet function. However, many recent studies have aimed to elucidate the direct effects of TZDs on platelet function. Our lab was the first to show that human platelets and megakaryocytes express functional PPARγ (55). The PPARγ ligands 15d-PGJ_2_ and rosiglitazone potently inhibit thrombin-induced CD40L surface expression and thomboxane B_2_ (TXB_2_) production and dampen ADP-induced aggregation (56). Other studies have confirmed these findings and further demonstrated that rosiglitazone and 15d-PGJ_2_ inhibited collagen-induced aggregation and prevented P-selectin exposure *in vitro* and *in vivo* (54, 57). Utilizing the specific PPARγ antagonist, GW9662, these effects were partially mediated through PPARγ in platelets from healthy donors (58). Moreover, pioglitazone was protective in a mouse model of thrombosis (57, 59). Similarly, using platelets from type II diabetics, which are hyper-responsive to agonist, rosiglitazone reduced aggregation and mediator release (36, 60). These data support the hypothesis that TZDs can regulate platelet function by directly acting on platelet PPARγ.

Interestingly, PPAR-independent pathways are evident upon treatment with some ligands. 15d-PGJ_2_ is an electrophilic compound that is known to form adducts with other cellular proteins, and could explain some of the PPAR-independent effects (61). Most interestingly, the mechanism of troglitazone differs from that of the structurally similar TZD, pioglitazone, in platelets. Although troglitazone and pioglitazone decreased platelet activation *in vivo*, only troglitazone directly inhibited platelet aggregation *in vitro* (62). However, in this study, only 1 μM of each TZD was examined for their effects on platelet function. In some cell systems, troglitazone is more potent than pioglitazone, despite having a higher EC_50_ for binding PPARγ and this may also be the case in platelets (63). It is possible that higher concentrations of pioglitazone would exhibit similar effects as troglitazone. Another possibility is that there may be PPAR-independent effects or differential signaling of PPARγ in human platelets. Clinical data points to some PPAR-independent actions of TZDs as pioglitazone has been shown to decrease the risk of myocardial infarction and stroke in type II diabetics, while rosiglitazone had no effect and may actually increase the relative risk (64).

## Signaling Mechanism of PPARγ in Platelets

Differential signaling of PPARγ is not an unprecedented finding, as PPARγ is known to recruit various co-activators after stimulation with different ligands. Although most TZDs bind identical binding pockets in PPARγ, their biological profiles are distinct (65, 66). This is in part due to differential recruitment of co-activators, but also possibly due to variations in availability of cofactors. In cell-based systems, PPARγ ligands can act as partial agonists in some cell types and full agonists in others (67). Additionally, different PPARγ ligands can recruit different co-activators in the same cell type, leading to different outcomes (68–70). These differences likely explain many of the adverse effects observed with some TZDs in clinical trials. Although still poorly understood, differential binding and recruitment of cofactors may explain the complex and sometimes contradictory actions of PPARγ in platelets, although no studies have evaluated this role of PPARγ in platelets to date.

**Figure 2:**
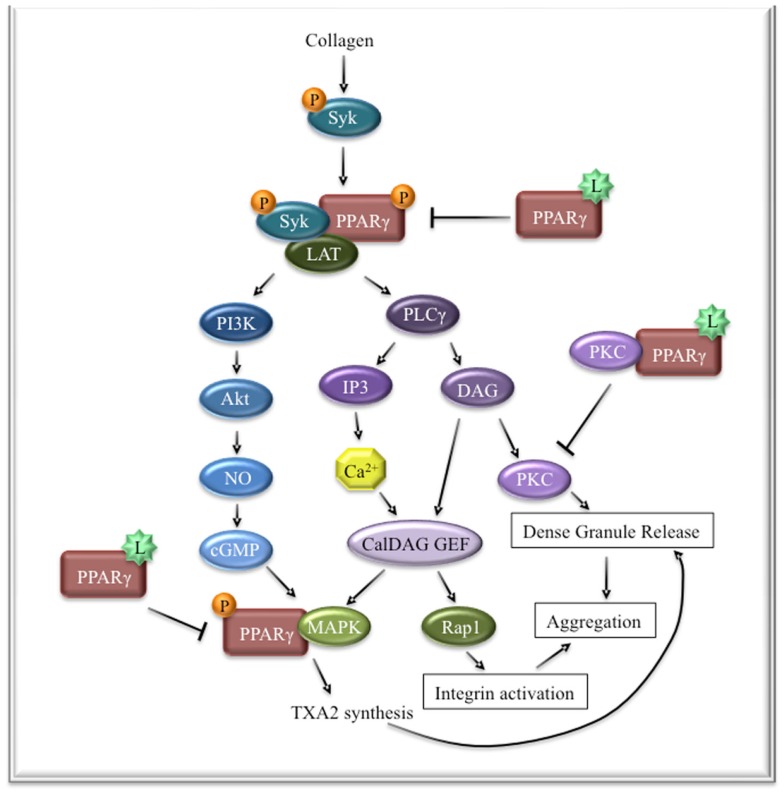
**The role of PPARγ in collagen signaling in platelets**. GPVI stimulation with collagen leads to the recruitment and activation of Syk. Syk recruits and phosphorylates PPARγ, which in turn leads to the recruitment of LAT, resulting in enhanced signal transduction downstream of GPVI. PPARγ ligands prevent the interaction of PPARγ with the Syk signaling complex, which is necessary for enhanced signaling of Syk and LAT. Ligand-bound PPARγ can also interact with PKCα, preventing it from mediating dense granule release. Lastly, ligand-activated PPARγ prevents PPARγ phosphorylation and interaction with MAPK, an essential downstream mediator of collagen signaling.

Although a transcription factor, PPARγ has been shown to serve many other important non-genomic roles in platelets and nucleated cells (Figure 2). Specifically in platelets, collagen stimulation results in PPARγ recruitment to the GPVI signalosome and interacts with the adapter molecule, Syk (71). As a result, linker of activated T cells (LAT) is recruited and forms a complex with Syk and PPARγ. In this setting, PPARγ appears to be necessary for enhancement of GPVI-mediated activation as treatment with PPARγ antagonists only partially blocks phosphorylation of LAT and the downstream targets phospholipase Cγ (PLCγ), PI3K, and Akt. Interestingly, several other groups have demonstrated that cytosolic platelet PPARγ is released into the supernatants and in platelet-derived microparticles rapidly upon activation, leaving very low levels of PPARγ in the activated platelets (34, 72). This may suggest that early signaling roles of PPARγ include recruitment to the LAT/Syk signaling complex, which results in its packaging and export into microparticles, possibly due to its proximity to the cell membrane. Alternatively, there may be different pools of PPARγ that could have different subcellular localization patterns. It is not known whether platelets contain an unknown endogenous PPARγ ligand that plays a role in this signaling pathway. However, exogenous treatment of activated platelets with PPARγ ligands inhibit its interaction with the LAT/Syk signaling complex and reduces the amplitude of the signal, thus reducing aggregation and mediator release. Similarly, PPARγ ligands also blunt release of PPARγ into the supernatants and platelet-derived microparticles (34).

Separate studies have suggested that PPARγ interacts with ERK and p38 MAPK, which are common downstream mediators of platelet activation, leading to granule secretion (73). Stimulation of human platelets with collagen led to phosphorylation of PPARγ and its subsequent association with p-ERK and p38 MAPK. Treatment with PPARγ ligands prevented these interactions, reducing granule release. Similarly, PPARγ has been shown to interact with PKCα in platelets in response to PPAR agonists, consequently reducing the activation of PKCα (74). Although complex, there is sufficient evidence to suggest that PPARγ plays an important role in platelet signaling. PPARγ knockout mice are embryonic lethal; thus, a megakaryocyte and platelet-specific PPARγ knockout mouse model would be invaluable in determining the biologic role of PPARγ in platelets. As platelets do not contain a nucleus, any genomic deletion of PPARγ in platelets must also be deleted in the parent megakaryocyte. Therefore, changes in platelet function in these mice could be due to the loss of PPARγ in the platelet or megakaryocyte. Our group has recently shown that overexpression of PPARγ in a megakaryocyte cell line resulted in enhanced platelet production upon stimulation with a PPARγ ligand (72). These data suggest that PPARγ may play an important role in platelet production from megakaryocytes and could potentially convolute findings from megakaryocyte and platelet-specific PPARγ knockout mice.

## PPARβ/δ

PPARβ/δ is broadly expressed and plays an important role in skeletal muscle where it regulates cellular proliferation, differentiation, and fatty acid catabolism (75). Similar to PPARγ, PPARβ/δ is found in human and mouse platelets and PPARβ/δ ligands inhibit platelet function (76). Treatment with the specific PPARβ/δ ligand, GW501516, significantly increased cAMP levels in mouse platelets and prevented aggregation (77). Utilizing platelets from PPARβ/δ knockout mice, the increase in cAMP was shown to be specific to PPARβ/δ signaling, as cAMP levels in knockout platelets were drastically lower in response to ligand. Interestingly, no difference in aggregation was observed between knockout and control platelets treated with GW501516, suggesting that the inhibition of platelet aggregation by GW501516 occurs independent of PPARβ/δ. Furthermore, no differences were observed between knockout and control platelets’ ability to produce cAMP or aggregate in response to ADP in the absence of PPARβ/δ ligand. This indicates that PPARβ/δ in platelets may require ligand binding to exert its inhibitory effects on platelet function. Nevertheless, these data demonstrate that PPARβ/δ is capable of signaling in platelets, although the physiologic relevance remains unclear at this point.

One possible mechanism by which PPARβ/δ dampens platelet activation may involve the endogenous antithrombotic molecule, prostacyclin I_2_ (PGI_2_). PGI_2_ is produced by endothelial cells and is reported to be a ligand for PPARβ/δ (78), although this has not been proven in platelets. Previous studies have shown that PGI_2_ can synergize with endothelial-derived NO to inhibit platelet aggregation in response to variety of agonists through a mechanism involving increased cAMP (76). Another possible mechanism of action of PPARβ/δ in platelets may involve modulation of PKCα function. Treatment of human platelets with GW501516 induced association of PPARβ/δ with PKCα in a dose-dependent manner (77). PKCα has been shown to positively regulate platelet adhesion, a function that was dampened with PPARβ/δ activation. Overall, very little is known about the biological significance and mechanism of PPARβ/δ in platelets, although the available data suggest a role in inhibiting platelet activation.

## PPARα

PPARα is a major regulator of fatty acid homeostasis and inflammation. It is highly expressed in brown adipose tissue, liver, kidney, heart, and skeletal muscle (75). PPARα agonists are used as a treatment for elevated plasma lipid levels and for their ability to increase the uptake of fatty acids and improve the high-density lipoprotein (HDL) to low-density lipoprotein (LDL) ratio. Little is known about the function of PPARα in regulating hemostasis, but its presence was recently discovered in platelets. Statins and fibrates are PPARα agonists and are widely prescribed for the prevention of coronary artery disease and atherosclerosis, thus reducing the risk of thrombotic events (74). In addition to their effects on lipid metabolism, statins and fibrates have been shown to activate PPARα (79). Specifically, fenofibrate decreased agonist-induced platelet activation and increased bleeding times in mice (74). Fenofibrate’s ability to inhibit platelet aggregation was abolished in the presence of a specific PPARα antagonist. Additionally, bleeding times in PPARα knockout mice were not affected by treatment with fenofibrate, unlike control mice. Interestingly, the baseline bleeding times in PPARα knockout mice were longer than control mice, suggesting an additional role for PPARα in maintaining hemostasis. Due to the usage of PPARα global knockout mice, it is unclear whether this difference was due to platelet-specific or multivariate effects. Although the mechanisms of action of PPARα in platelets have not been thoroughly investigated, some evidence suggests that it may regulate PKCα activity, similar to PPARγ and PPARβ/δ (80).

## Non-Genomic Functions of PPARs: Lessons from Nucleated Cells

The wide use of PPAR ligands in the clinic has led to the discovery of numerous pleotropic effects of these compounds. These include PPAR-dependent, non-genomic actions in addition to PPAR-independent signaling of ligands (81, 82). The PPAR-independent signaling mechanisms of PPAR ligands will not be discussed here, but these effects must be kept in mind when interpreting data investigating the effects of PPAR ligands on platelet function. In fact, these likely play an important role in regulating platelet function, perhaps through affecting mitochondrial activity, as many effects of PPAR ligands in platelets cannot be reversed using PPAR antagonists. Additionally, many PPAR ligands, such as unsaturated fatty acids, can cross-react with all PPAR isoforms at higher concentrations, further complicating the interpretation of these findings (83). It may be possible to generate useful hypotheses about how PPARs signal in platelets based upon similar studies in nucleated cells.

## PPARγ–PKCα Crosstalk

In human epithelial colorectal adenocarcinoma cells, PPARγ has been shown to regulate NFκB activity in a non-genomic fashion involving their direct association (84). In this model, PPARγ bound to p65 in the nucleus, resulting in export of both proteins to the cytoplasm. Consequently, cytoplasmic localization of NFκB prevented its proinflammatory transcription-dependent effects. It is possible that PPARγ can also physically interact with p65 in platelets. This interaction could inhibit NFκB activity in platelets, which would exert anti-inflammatory and anti-platelet effects. PPARγ has also been shown to form a direct interaction with PKCα upon stimulation with various PPARγ ligands in macrophages (85). The physical association of PKCα with PPARγ prevented PKCα translocation to the membrane and subsequent degradation. However, in this system PPARγ was only able to negatively regulate PKCα activation in response to low-dose PMA stimulation. This suggests that the inhibitory effect of PPARγ can be overridden with stronger stimulation.

## PPARα–PKCα Crosstalk

**Figure 3:**
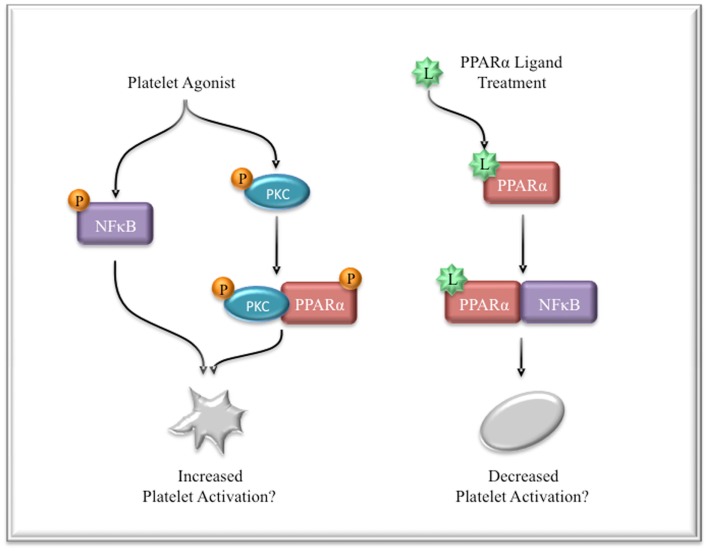
**Possible mechanism of PPARα and NFκB regulation in platelets**. Platelet activation leads to NFκB phosphorylation and activation, which potentiates activation signals. PKCα is also phosphorylated and activated in response to stimulation. This may lead to an interaction between PPARα and PKCα, leading to increased activation. Treatment with PPARα ligands prevents PPARα binding to PKCα, but instead enhances its interaction with NFκB, thus functionally sequestering PPARα and NFκB from participating in activation signaling.

Interestingly, PPARα has also been shown to directly interact with PKCα in macrophages (86). In response to LPS stimulation, PKCα bound and phosphorylated PPARα. However, in the presence of the PPARα ligand, simvastatin, PPARα no longer bound PKCα and consequently was able to transrepress NFκB activation. In this model, PKCα likely acts to deactivate PPARα’s inhibitory actions on the proinflammatory NFκB signaling. This may represent a mechanism by which PPARα ligands, such as statins, exert potent and rapid anti-inflammatory actions. Although the interaction of PPARα with PKCα was postulated to negatively regulate PPARα’s function in this system, it is possible that this complex serves additional and possibly proinflammatory functions. Similarly, these data suggest that in platelets, ligand-induced activation of PPARα may function differently than phosphorylation-induced activation, resulting in different or possibly contradictory actions. This may even explain the discrepancy between the bleeding phenotype observed in PPARα knockout mice, while control mice also had increased bleeding times with PPARα activation. Perhaps, PPARα, independent of ligand, acts as a positive regulator of platelet hemostatic function by enhancing activation of PKCα, while ligand-activated PPARα sequesters and inhibits NFκB thus, negatively regulating platelet activation (Figure 3).

## PPARγ and PPARα Regulate MAP Kinases

Another possible role of PPARα in platelets involves regulation of MAP kinases. In T cells, PPARα was shown to inhibit p38 MAPK activation, but only in the absence of ligand (87). Additionally, this regulation of p38 MAPK appeared to be independent of direct interaction with PPARα, but likely required an unknown secondary mediator. Activation of p38 MAPK occurs early in platelet activation, albeit transiently, to induce granule release that stimulates the second wave of aggregation (88, 89). Subsequent ligand binding to αIIbβ3 downregulates active p38 MAPK (90). Perhaps, PPARα mediates the regulation of p38 MAPK in these conditions or possibly under shear stress, whereby p38 MAPK mediates adhesion in flow.

PPARγ has also been shown to regulate MAPK pathways in nucleated cells through non-genomic mechanisms (91). PPARγ reversibly interacts with MEK1 via its AF2 domain, resulting in export from the nucleus to the cytoplasm. This corroborates evidence suggesting that PPARγ ligands regulate downstream ERK signaling (92). Interestingly, PPARγ activation led to rapid ERK phosphorylation in human prostate cancer cells, vascular smooth muscle cells, and human microvascular endothelial cells, but inhibited ERK activation in adrenocortical cancer cells (93–95). Similarly, PPARγ enhanced ERK activation in rabbit renal cortex cells, but had no effect in mouse cells (96). These data suggest cell type-specific and species-specific effects of PPARγ, possibly through availability of different co-activators. The effects of PAPRγ activation on the MEK/ERK pathway in platelets has not been investigated, but represents a promising avenue of further research.

## Liver X Receptors

**Figure 4:**
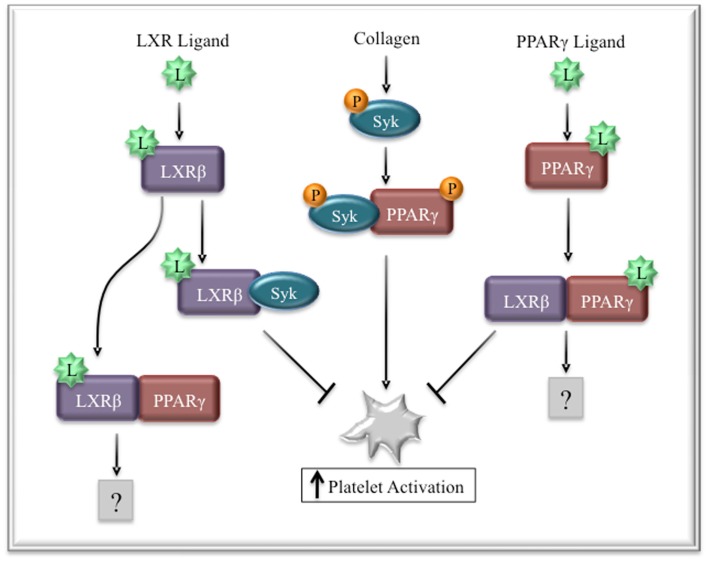
**Regulation of collagen signaling by LXRβ and PPARγ**. Collagen signaling leads to the phosphorylation of Syk and PPARγ. Ligand-activated LXR binds Syk, preventing its interaction with PPARγ, thus decreasing platelet activation. Similarly, PPARγ ligands or LXR ligands can induce the interaction of PPARγ and LXR, reducing the availability of PAPRγ to participate in collagen signaling.

Liver X receptors (LXRs) are transcription factors that play key roles in cholesterol homeostasis by regulating genes, such as apolipoprotein E and cytochrome P450 7α-hydroxylase 1 (Cyp7a1) (97, 98). Recent studies have demonstrated that platelets express LXRβ and its ligands inhibited collagen-induced aggregation (99). Thrombin and fibrinogen-induced activation was affected to a lesser degree, requiring high doses of LXR ligands to exert comparable inhibitory effects. Collagen-induced Syk phosphorylation was strongly inhibited by pretreatment with LXR ligands, while LAT and PLCγ phosphorylation were inhibited to a lesser degree, which could be explained by the high concentration of collagen used for activation (50 μg/mL). Interestingly, and opposite of the results seen with PPARγ (71), LXR ligands induced association of LXR with Syk and PLCγ. Moreover, stimulation of platelets with PPARγ or LXR ligands led to an association between the two transcription factors, which was diminished with higher concentrations of ligand. Taken together, these data suggest that LXR and PPARγ can physically interact in platelets and this may represent a novel regulatory mechanism of collagen-induced activation (Figure 4). In cell free systems, LXRβ was able to bind all three PPAR isoforms with different affinities (100). Moreover, different PPAR ligands altered the affinities of these interactions. For example, troglitazone inhibited PPARγ/LXRβ interactions, while GI262570, a PPARγ ligand with high binding affinity, had no effect on this interaction. This may help to explain why different PPAR ligands exert different effects on platelet signaling and function.

## Retinoid X Receptors

Retinoid X receptors comprise a group of nuclear receptors that recognize vitamin A metabolites (101). The three different isoforms of RXRs (α, β, and γ) are expressed in different tissues, with RXRα and RXRβ being fairly widely expressed and RXRγ is mainly expressed in skeletal muscle and heart tissue (102). RXRα is the heterodimeric binding partner of PPARγ, which is essential for mediating the genomic effects of PPARγ. Platelets express the RXRα and β isoforms, but not RXRγ, and RXRs immunoprecipitate with PPARγ in platelets. (103) Similar to PPARγ ligands, the RXR ligand, 9-cis-retinoic acid (9cRA), inhibited agonist-induced platelet activation. Interestingly, however, 9cRA preferentially inhibited ADP and U46619 (a thromboxane mimetic)-induced aggregation, but did not alter collagen-induced activation. This is surprising in light of the fact that PPARγ ligands dampen signaling through the collagen receptor (Figure 2), suggesting that RXR in platelets can signal non-genomically independent of PPARγ. RXRα co-immunoprecipitated with Gq11 in resting platelets and this interaction was enhanced when platelets were stimulated with 9cRA. In this manner, ligand-bound RXRα may sequester Gq from its traditional role in propagating signals from G-protein coupled receptors, such as the ADP and thromboxane receptors.

## Glucocorticoid Receptors

The glucocorticoid receptor (GR) is a nuclear hormone receptor that binds glucocorticoids, such as dexamethasone and prednisolone, resulting in an anti-inflammatory response (104). Human platelets express GR and differentially respond to its ligands (105). One study found that human platelets were less responsive to activation when treated with prednisolone, but not dexamethasone (106). Furthermore, the inhibitory effects of prednisolone on platelet function could be prevented by treatment with the GR antagonist, RU486. Interestingly, binding assays in human platelets revealed that both glucocorticoids could bind GR and increase its association with the chaperone protein, HSP90. In nucleated cells, ligand binding to GR causes HSP90 dissociation from the GR complex, which reveals its nuclear localization signal (107). However, the opposite effect was found in platelets, suggesting a different method of regulation. Moreover, a unique dimerization was identified in human platelets between GR and the mineralocorticoid receptor (MR), which can bind some of the same ligands as GR, such as dexamethasone. This may help to explain why dexamethasone affected platelet function differently from the more specific GR agonist, prednisolone. Additionally, mineralocorticoids activate PI3K, consistent with the finding that mineralocorticoid actions are associated with increased risk of vascular disease (108).

## Aryl Hydrocarbon Receptor

The aryl hydrocarbon receptor (AHR) is a well-known toxicant-sensing receptor that also plays essential roles in immune function and hematopoiesis (109). Common AHR ligands include polycyclic aromatic hydrocarbons, lipoxin A4, and 2,3,7,8-tetrachlorodibenzo-*p*-dioxin (TCDD). Mouse platelets express AHR, which is known to play an important role in hematopoiesis and megakaryocyte development (110). Platelets from AHR knockout mice exhibit defective collagen signaling, with inhibited collagen-induced aggregation and spreading. Of note, platelets from AHR knockout mice had lower levels of the collagen receptor, GPVI, and undetectable levels of Vav proteins. Vav1 and Vav3 are activated downstream of many signaling pathways in platelets, and play a role in responding to collagen (111). Although the defects in platelet collagen signaling in AHR knockout mice are intriguing and may suggest an important role for this transcription factor in platelet biology, a more thorough investigation is needed. It is still unknown whether platelets respond to AHR ligands and whether they affect agonist-induced activation. Moreover, it is possible that the defects in collagen signaling in AHR knockout mice could be solely attributed to defective thrombopoiesis and altered platelet composition. Limited studies have evaluated the effects of AHR ligands in hemostasis. Polychlorinated biphenyls, which are non-specific AHR ligands, enhanced human platelet activation (112). Additionally, the specific AHR ligand, TCDD, was shown to induce thrombocyte aggregation in zebrafish (113). This can likely be attributed to its non-genomic actions in thrombocytes, as these effects were seen as early as 30 min post-treatment. Further research investigating the effects of AHR activation in platelets may reveal novel actions of environmental toxicants on hemostasis and cardiovascular risk.

The AHR has been shown to form a complex with the p65 subunit of NFκB in breast cancer cells (114). Furthermore, this interaction was found to be functionally relevant, resulting in proliferation and tumorigenesis. Additional roles for the AHR in regulation of NFκB signaling have been shown by our group in fibroblasts (115, 116). We found that absence of the AHR coincided with decreased expression of the non-canonical NFκB member, RelB. Signal-specific phosphorylation of RelB targets it for proteosomal degradation. It is possible that physical association between the AHR and RelB prevents its proteosomal degradation and allows it to exert anti-inflammatory effects, similar to its regulation in T cells (117).

## Signal Transducer and Activator of Transcriptions

The signal transducer and activator of transcription (STAT) family of proteins were some of the first identified functional transcription factors in platelets (118). Human platelets express STATs 1, 3, and 5, which traditionally play a role in transducing signals from cytokine receptors to elicit an immune response (119). Early on, it was known that thrombopoietin could act on human platelets and this resulted in STAT3 phosphorylation, although the biological significance was unclear. Recently, the role of STAT3 in platelet signaling has been investigated more thoroughly, uncovering an important role for it in GPVI signaling (120). Pharmacologic inhibition of STAT3 signaling or dimerization resulted in a hyporesponsive phenotype to low-dose collagen activation. Additionally, platelets from platelet and megakaryocyte-specific STAT3 knockout mice had lower levels of collagen-induced aggregation, decreased P-selectin expression, and slowed thrombus formation. Interrogation of the signaling cascade revealed that STAT3 was activated by JAK2, in the same manner as in nucleated cells. Traditionally, STAT3 homodimerizes upon phosphorylation by JAK2, then translocates to the nucleus where it acts as a traditional transcription factor. However, in platelets, phosphorylation of STAT3 by JAK2 resulted in dimerization and activation of PLCγ. Syk was found to be an upstream activator of this pathway and was complexed with STAT3 and PLCγ after activation with collagen (121). These data suggest that STAT3 plays an important regulatory role in platelet activation downstream of collagen and thrombopoietin signaling.

## The Divergent Roles of Transcription Factors

The investigation of transcription factors in platelets has often led to convoluted and sometimes contradictory findings. This can most clearly been seen in the evaluation of the roles of PPARα in platelets. In this case, PPARα appears to play an important role in platelet activation, but has the opposite effect in the presence of a PPARα ligand (74). Additionally, phosphorylated PPARγ positively regulates collagen signaling, while ligand-bound PPARγ inhibits activation (71). These data support the hypothesis that ligand-induced activation of transcription factors can function differently than phosphorylation-induced activation (Figure 3).

## Microparticles as Transports of Transcription Factors

Microparticles are plasma membrane-derived vesicles ranging in diameter from 0.1 to 1 μm that are present at levels of approximately 5–50 μg/mL in blood plasma (122). Platelets and megakaryocytes are the primary source of microparticles in the blood circulation (about 80%), whereas other microparticles are derived from erythrocytes, endothelial cells, and granulocytes (122–124). Since their discovery, the role of platelet microparticles in coagulation was evident, and later was supported by identification of tissue factor expression (123) and a phosphatidylserine-rich outer membrane (125) that binds coagulation factors to aid in their assembly and enzymatic processing. Microparticles are produced from resting cells, during apoptosis or during cell activation, likely resulting in different internal and surface composition. Microparticles were shown to differ in composition between human samples and between microparticle size classes (126, 127). Packaging mechanisms for microparticles have not yet been identified and such studies are crucial for the understanding of microparticle influences on their environment. Whether microparticle packaging is a passive or active process, increased proximity of mediators to the cell plasma membrane would likely increase their chances of becoming encapsulated by the released microparticles.

## Microparticle Functions and Roles in Inflammation

Microparticles are postulated to have several means to influence their environment. Burger et al. described their ability to (1) promote coagulation, (2) scavenge NO, (3) generate reactive oxygen species, (4) cleave cellular surface proteins via metalloproteinases, (5) signal cells via surface proteins, and (6) deliver cargo via transfer of membrane and internal contents (128). Additionally, microparticles are postulated to contribute to, and sometimes exacerbate inflammation. It is likely that surface interactions and the less studied delivery of internal microparticle contents are both contributing to the influences of microparticles on their environment.

## Transcellular Communication

Circulating microparticles can interact with other blood cells they encounter, such as leukocytes, lymphocytes, platelets, and with endothelial cells. Specific modes of cell–microparticle interactions include surface receptor signaling, plasma membrane fusion, or internalization of microparticles (129). The fusion of platelet microparticle membranes to target cell membranes was demonstrated to transfer the surface protein CXCR4 to cells causing recipient cell susceptibility to human immunodeficiency virus infection (130). Membrane fusion or internalization of microparticles could also cause microparticle internal composition to be transferred into the recipient cell cytoplasm. Lipids (131), RNA, and protein (129) have been seen to be delivered in this fashion, and multiple examples of each are reviewed by Mause and Weber (132). Arachidonic acid is a lipid mediator delivered by platelet microparticles that can be further processed into thromboxane A_2_ by recipient platelets and contribute to their activation (131). Overall, platelet microparticles contain various mediators that can be delivered to surrounding cells to impact their function.

## Transcription Factors in Microparticles and Roles in Transcellular Communication

The influence of microparticles on recipient cell function is based on microparticle composition. Not surprisingly, blood microparticle protein composition was found to be highly variable between healthy humans (127). Our work showed that platelet microparticles contain transcription factors, such as PPARγ, derived from parent cells (34). Furthermore, proteomic analysis has led to the discovery of three other transcription factors in platelet microparticles, RuvB-like 2, STAT3, and STAT5A (133).

**Figure 5:**
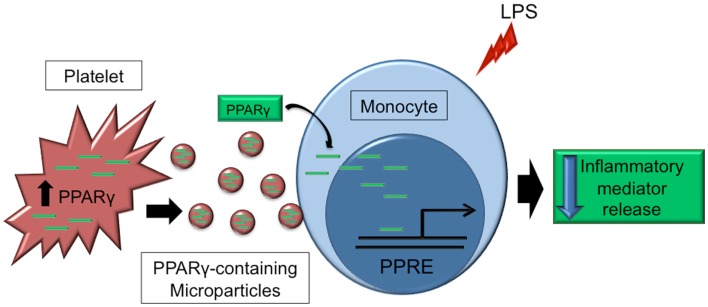
**Platelet microparticles as a method of transcellular delivery of transcription factors**. Platelet-derived PPARγ is packaged into platelet microparticles, which can deliver intact PPARγ protein to target cells, such as monocytes. PPARγ can then bind to peroxisome proliferator response elements (PPRE) in the nucleus to affect transcription of target genes. Transfer of PPARγ to monocytes via microparticles has been shown to decrease LPS-induced inflammatory mediator release.

Transcription factors are transported from cells through microparticles and retain function within the recipient cells (34, 72). Our lab was the first to show this ability with platelet-derived microparticles delivering functional PPARγ to THP-1 monocytes, detected through an electrophoresis mobility gel shift assay (134). We have since developed a novel platform technology to engineer microparticles through overexpression of PPARγ in platelet and megakaryocyte-derived microparticles obtained from the cultured megakaryoblastic cell line, Meg-01 cells. We showed that these engineered microparticles could be taken up by THP-1 monocytes, and that the transferred PPARγ was functional within recipients shown by induction fatty acid binding protein-4 (FABP4) expression, a unique PPARγ-specific target gene (72) (Figure 5).

To identify the significance of the transferred PPARγ to recipient cells, we compared recipient cell responses incubated with microparticles that did or did not contain PPARγ, to inflammatory stimuli (135). Monocytes that received PPARγ-containing microparticles had decreased inflammatory mediator production compared to the control microparticles. PPARγ activation has been shown to induce cell differentiation (136, 137). Our work also supported the influence of PPARγ on monocyte differentiation as the cells receiving PPARγ-containing microparticles became more adherent through increased integrin expression and fibronectin production (135). These data support the notion that circulating microparticles could have a profound impact on inflammatory cells during responses to insult and injury. Specifically, the anti-inflammatory PPARγ and proinflammatory NF-κB composition of microparticles could vastly dampen or heighten responses of recipient cells.

## Diagnostic and Therapeutic Potential of Microparticles

Measurements of microparticle abundance are becoming a more popular assessment of systemic inflammation. With current technology, it is easier and less expensive to measure microparticle number and surface composition rather than running entire microparticle proteomic studies with each patient sample. Microparticle number and surface phenotype have provided descriptive diagnostic tools indicative of disease severity. However, evaluation of the total protein composition of microparticles would tell an infinitely more detailed summary of patient health status. Particularly, looking at levels of the highly influential transcription factors within circulating microparticles would provide insight into the inflammatory state from the cells generating the microparticles as well as the potential inflammatory impact on recipient cells within the blood.

Delivery of platelet proteins such as PPARγ could have an anti-inflammatory and/or pro-differentiation influence on activated cells via microparticles and act as a physiological means of quenching inflammation. Transcription factors would be among the most influential mediators to deliver to a cell, as they are capable of initiating transcripts of multiple downstream products that could influence numerous networks and pathways. Certain transcription factors may require signal or ligand activation to fully function in a microparticle delivery setting. In the case of PPARγ, endogenous ligands such as 15d-PGJ_2_ and others may be present or synthesized. Whereas the components delivered by microparticles may be short-lived, their effects on the recipient cells may sometimes be permanent, such as inducing irreversible differentiation or death of recipient cells.

Overall, platelet microparticles are abundant and influential transcellular vesicles. They contain proteins, lipids, and RNA derived from their parent cells. Therefore, these circulating biomarkers provide insight into the cumulative activation and inflammatory state of all blood cell populations. Importantly, microparticles have repeatedly been shown to not just be cell byproducts but rather influential delivery mechanisms. Transportation of transcription factors to cells could influence several ongoing or initiate new pathways causing profound impacts within recipient cells. Transcellular communication involving transcription factors via platelet microparticles substantiates another possible key role of transcription factors presence in platelets.

## Conclusion

Transcription factors play numerous important and previously unrecognized roles in regulating platelet function in a non-genomic manner. Additionally, transfer of intact and functional transcription factors to other cells via microparticles may serve as a novel regulatory mechanism for inflammation. The initial discovery of transcriptional regulatory elements in a particular protein does not exclude the possibility of non-genomic roles for that protein as well. The anucleate platelet can serve as an important model system to study non-genomic roles of transcription factors. Further elucidation of the non-genomic functions of transcription factors will yield important discoveries that have the potential to illuminate platelet biology and also help to explain the pleotropic effects of pharmaceuticals that target these pathways.

## Conflict of Interest Statement

Neil Blumberg has received lecture honoraria and consulting fees from Antek, Inc., Fenwal, Pall BioMedical, and Caridian (Terumo), manufacturers of leukoreduction filters, blood component equipment, and cell washing devices. The other authors have nothing to disclose.
